# A Low-Cost Priapism Detumescence Simulator for Emergency Medicine Residents

**DOI:** 10.7759/cureus.15782

**Published:** 2021-06-20

**Authors:** Zach Hampton, Nickolas Meier, John Casey

**Affiliations:** 1 Emergency Medicine, OhioHealth Doctors Hospital, Columbus, USA

**Keywords:** ischemic priapism, simulation in medical education, emergency medicine resident, teaching in emergency medicine, low-cost task trainers

## Abstract

Introduction: Ischemic priapism is an emergent condition requiring immediate intervention. However, the incidence is estimated to be very low. Given the low incidence of this pathology, some emergency medicine residents do not have the opportunity to perform needle aspiration, the critical procedure to achieve detumescence. We sought to fill this void by creating low-cost, high-fidelity trainers for emergency medicine resident procedural competency.

Methods: Using items obtained online and through our hospital’s simulation department, we created a low-cost priapism trainer from previously described literature. Residents completed a lecture, lab, and short post-course survey regarding helpfulness, realism, prior procedure experience, and future applicability of our training device. Descriptive data were calculated using the median with interquartile range.

Results: The trainer cost roughly $25 to create per unit. All participants rated the trainer a 5 for helpfulness. When asked if the lab appeared realistic, there were overly positive responses with a median of 5 (interquartile range [IQR] 4-5), with every respondent selecting either realistic (4 on the Likert scale) or very realistic (5 on the Likert scale). All participants (100%) agreed that they would recommend the use of this trainer for future medical students and residents.

Conclusion: Priapism, specifically ischemic priapism, is truly an emergent condition requiring immediate intervention. The incidence of this condition is low, and some emergency medicine residents may not have the opportunity to perform the procedure during training. Given the need for simulated experiences, we developed a low-cost, high-fidelity trainer that was found to be helpful and realistic to emergency medicine residents. While other models exist, our model minimizes cost while maximizing realism.

## Introduction

Priapism is a persistent and painful erection lasting longer than four hours without sexual stimulation. Priapism is classified into three subtypes: ischemic (low-flow), non-ischemic (high-flow), and stuttering priapism. Ischemic priapism is characterized by a persistent, painful erection with remarkable rigidity of the corpora cavernosa caused by a disorder of venous blood outflow from this tissue mass and is similar to penile compartment syndrome [1]. The ischemic cause is the most urgent, requiring immediate intervention.

The incidence of priapism is not fully known. Multiple studies have been completed, suggesting 0.3 to 1.0 per 100,000 males [2-4]. Ischemic causes are estimated to account for 95% of all cases [1]. Given the low incidence of this pathology, many emergency medicine residents may not have the opportunity to treat such patients while in training. Treatment of ischemic priapism includes phlebotomy and phenylephrine injection into the corpus cavernosa [1]. Procedural competency is integral to emergency medicine resident education. To help with mastery of rarely performed procedures, task trainers have become more prevalent within graduate medical education. Studies have shown increased procedural confidence with the use of task trainers [5]. Given the importance of this procedure within the scope of emergency medicine practice, there exists a need to develop a low-cost, high-fidelity trainer.

There is a paucity of options available commercially [6], but those that exist range from less expensive, low fidelity models, to more expensive, high-fidelity models [7,8]. Cost can often be prohibitive when using task trainers to educate a large cohort of residents. Our objective was to create a low-cost, high-fidelity priapism trainer for emergency medicine residents. Secondary objectives were to ensure that the trainer was beneficial to resident education, realistic, and appropriate for future use.

## Materials and methods

Design

The general design was inspired by previous work from Ruest et al., who used a condom trainer and penrose drains to build a more elaborate trainer [9]. The condom trainers used in their study were too cost-prohibitive for our design, so a more simple, less expensive condom trainer was used for our design. Our aim was to create a high-fidelity priapism trainer to be used for procedural education with emergency medicine residents. The model was built with the help and guidance of our simulation department. The cost of the condom trainer was approximately $17. Though there are many options available, we used a generic rubber and silicone-based condom trainer, which was purchased from Amazon. Unfortunately, our most recent search shows this model is no longer available for purchase. The smaller supplies, including penrose drains, butterfly needles, artificial blood, and syringes were obtained through our simulation department at no cost; however, the cost of these supplies are estimated in Table 1.

**Table 1:** Estimation of cost for supplies.

Item	Cost
¼” Penrose Drains (18 Inches)	$8-$15 ($2 per 6 inches)
Artificial Blood (Water with Red Food Coloring)	$2
Butterfly Needle (50 ct Box)	$78 ($2 per needle)
10 cc Syringe (5 Pack)	$7 ($1.40 per Syringe Reusable)

We estimate the cost per trainer is approximately $25. We built four trainers to be used for this educational activity. Considerations while building these trainers included ease of reproducibility, simple design, reusability, and relatively low cost.

**Figure 1:**
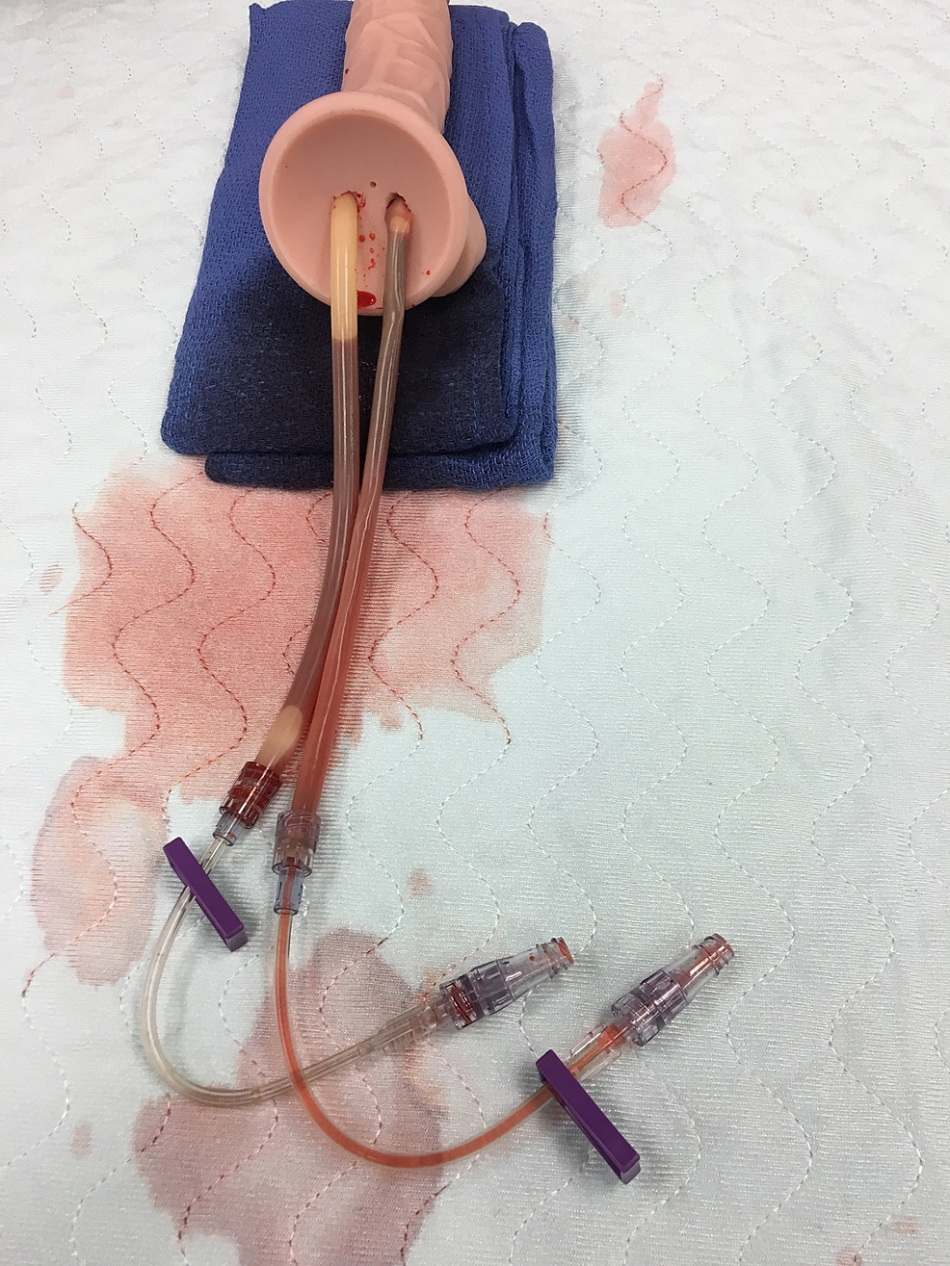
¼” drill, penrose, luer lock system

**Figure 2:**
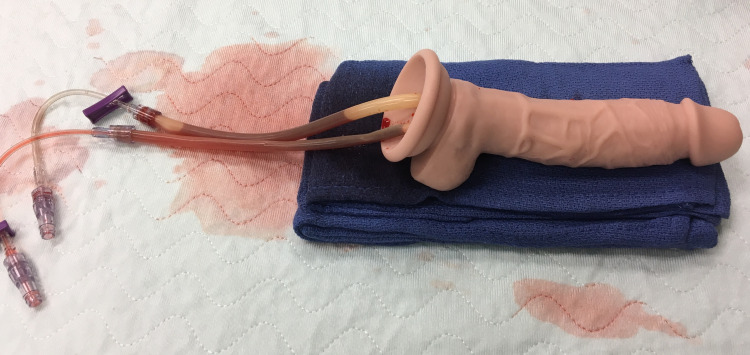
Final trainer. Luer locks allow more blood to be replaced in the penrose drains.

As shown in Figures 1 and 2, a ¼” drill was used to create two tunnels that traveled approximately ⅔ the length of the shaft. The tunneling was completed in such a fashion that there was a distinct left and right channel, slightly superior to the center of the coronal plane on the proximal segment of the penis. This design was used to represent the right and left corpora cavernosa, providing the ability for the user to access the penrose with a butterfly needle and aspirate blood from the erect penis. In the clinical setting, this same access would be used to inject small aliquots of phenylephrine as well, though this was not the main focus of the trainer. Using a bougie, the penrose drains were inserted into the predrilled holes and filled with artificial blood. Luer lock J loops were attached to the ends of the penrose drains by simply pushing the drains into the luer lock, which fit snuggly. This kept the artificial blood in the tubing and allowed for more blood to be placed into the drains after each demonstration. Since the condom trainer was made of rubber, it provided an autoseal after each needle insertion, allowing multiple uses without decreasing the fidelity of the trainer.

Implementation

**Figure 3:**
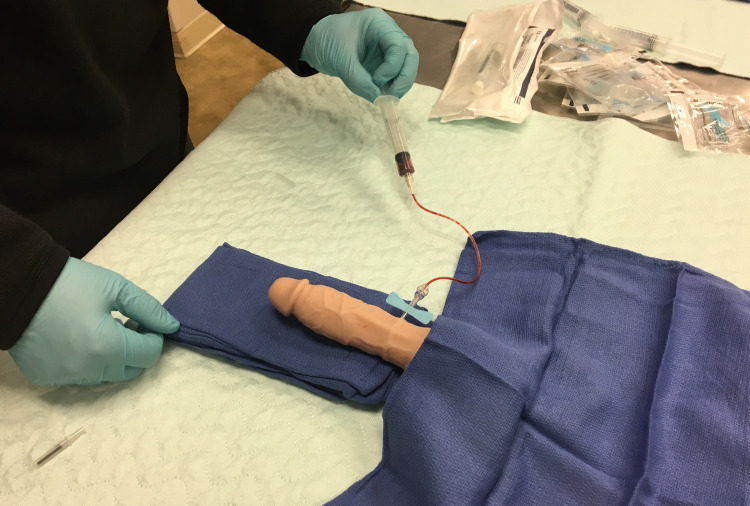
Model in use demonstrating the aspiration portion of the procedure.

The trainer was used for a single day simulation session with emergency medicine residents ranging from the post-graduate year (PGY)-1 to PGY-4. Residents were given a five-minute presentation on priapism, including an overview of how to perform needle aspiration and phenylephrine injection. After this short education session, residents were given approximately 15 minutes to work with the trainers (Figure 3). The residents present for the simulation (n=22) were divided into three smaller groups to ensure that all residents had an opportunity to adequately interact with the model. After the lab, each resident completed a short, anonymous survey in order to obtain feedback on the utility of the model. Survey domains included helpfulness, realism, prior procedure experience, and future applicability. Helpfulness and realism were assessed using a Likert scale from 1 (not helpful/not real) to 5 (very helpful/very real). These data were reported as median with interquartile range. The other two questions on the survey sought to determine how many residents had previously performed the procedure and whether they felt that the trainer should be used to educate medical students and residents in the future. These questions were tabulated as raw percentages.

## Results

**Figure 4:**
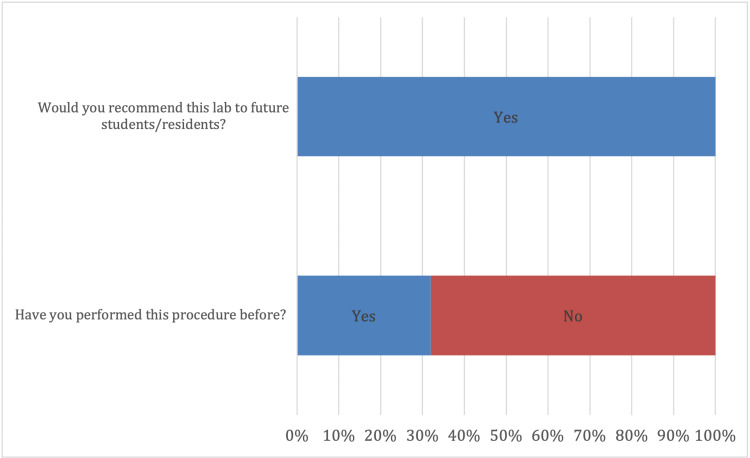
Experience and future use (n=22). All participants recommended the trainer for future use; 68% (n=15) had never performed this procedure.

**Figure 5:**
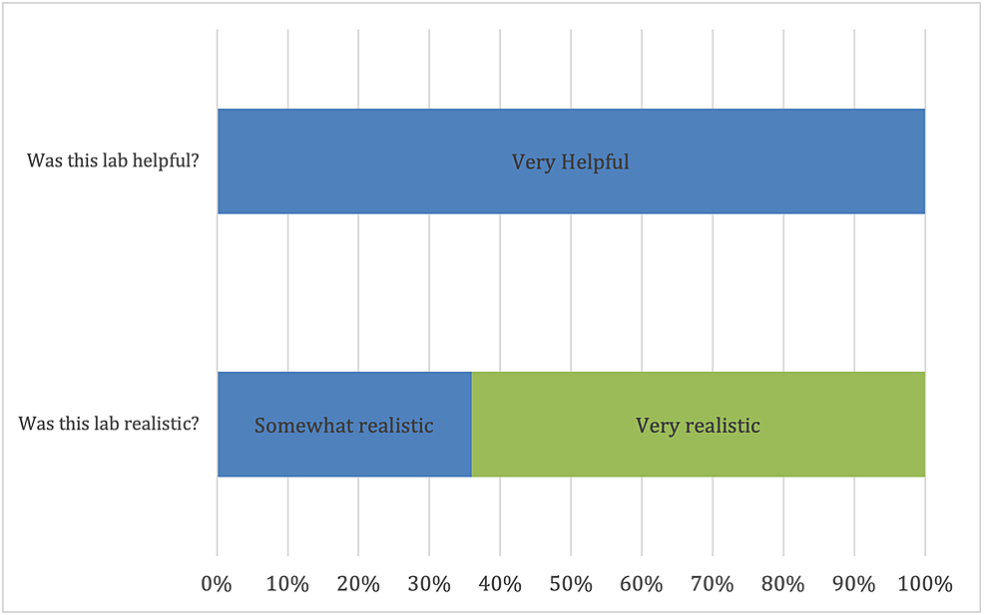
Survey results for helpfulness and realism. All participants (100%) felt that the lab was very helpful (5); 64% of participants felt the trainer was very realistic, while 36% felt it was somewhat realistic.

Our finished trainer was used for resident simulation with good effect. Data from our survey are presented in Figures 4 and 5. Twenty-two participants completed the training session. Of those, 68.18% had never attempted this procedure. All participants rated the trainer a 5 (interquartile range [IQR] 0) for helpfulness. When asked if the lab performed realistically, there were overly positive responses with a median of 5 (IQR 4-5). All participants responded as either somewhat realistic (4 on the Likert scale) or very realistic (5 on the Likert scale). All participants agreed that they would recommend the use of this trainer for future medical students and residents.

## Discussion

The results of our post-simulation survey were extremely positive, with all residents rating the trainer as “very helpful” and “somewhat realistic,” or “very realistic.” Prior experience with the procedure did not appear to change the perceived helpfulness or realism of the model. All residents recommended this trainer for future use with medical students and residents, which we believe is likely due to the low incidence of the procedure, leading to an inability to maintain procedural competency. Task trainers are used for many other low incident procedures (i.e., cricothyrotomy [10-12] and lateral canthotomy [13,14]) within emergency medicine, so it is not surprising that this trainer was viewed favorably under similar circumstances. Like other important emergency procedures, competency for emergency medicine residents should be considered of utmost importance, especially for those who will practice in areas without prompt urological consultation. 

There are several attributes that make our model ideal for future use. The creation of this trainer was relatively simple and, as outlined in the methods section, can be easily reproduced with only a few easily attainable supplies. The cost per trainer is also relatively low, estimated at only $25. This trainer can also be reused multiple times, as the trainer provides a self-seal after each aspiration and the penrose drains can be easily replaced with minimal added cost. There is no other cost for maintenance and the models can be stored without any special requirements. 

Despite the positive results of the trainer, we did receive valuable feedback from participants regarding possible improvements. Some of these comments included using a larger penrose drain to replicate a larger corpus cavernosum, as some participants had a difficult time finding the penrose drains while attempting to aspirate. Others asked for more equipment during the simulation. There are several ways to perform needle aspiration and phenylephrine injection. One way involves a three-way stopcock which allows a clinician to aspirate and inject through a single access line. While this is not necessarily pertinent to the design of the trainer, it is an easy change to improve the overall simulation.

As we discussed, our model was inspired by a previous model from Ruest et al. [9]. The authors used a similar, but hollow phallic model with larger penrose drains for the corpora cavernosa, as well as the corpus spongiosum. Their model was tested on a similar population of 49 emergency medicine residents, showing positive results across multiple domains and PGY levels. Several other publications have explored priapism trainers. Dai et al. used hotdogs and red vine candy to create a very low-cost trainer ($1.25), which showed promising results amongst junior level urology residents [7]. One limitation and concern for the authors was generalizability, particularly to emergency medicine learners. Recently, Chen et al. undertook a thorough systematic review on urological surgery simulation trainers [6]. While the authors looked at various products, including those outside the scope of priapism, they noted a wide range of prices from $1.25 (as discussed above) to $120 for higher fidelity models. We believe the relatively low cost of our model helps to fill a special niche within this line of products, one where lower cost is prioritized, but not at the expense of realism.

There are some limitations to the trainer. First, our sample size is low and limited to a single emergency medicine residency. We would need more participants to fully support the helpfulness and realism of our trainer. Second, we did not complete a pre-procedural survey, so we cannot comment on improvements in skill level or acquisition. Future studies should focus on expanding participants to include other resident specialties, medical students, advanced practice clinicians, and attendings within specialties that might perform this procedure. Fourth, though we believe our trainer to be high fidelity, certain aspects such as the diameter of the penrose drains and the inability to perform a dorsal penile block will limit the use of the trainer.

## Conclusions

Priapism, specifically ischemic priapism, is truly an emergent condition requiring immediate intervention. The incidence of this condition is low, and some emergency medicine residents may not have the opportunity to perform the procedure during training. Given the need for simulated experiences, we developed a low-cost, high-fidelity trainer to increase resident procedural competency. While this trainer was tested on a relatively low number of emergency medicine residents, results were favorable across all domains showing the need for further development of similar low-cost, high-fidelity trainers.
